# 307. Exploring HLA genotypes as contributing risk factors in Staphylococcus aureus bacteremia

**DOI:** 10.1093/ofid/ofad500.379

**Published:** 2023-11-27

**Authors:** Yazhong Tao, Frances Hung, Matthew Jogodnik, Dezhao Fu, Jackson L Kair, Derek M Dykxhoorn, Felicia Ruffin, William K Scott, Cliburn Chan, Vance G Fowler, Annette M Jackson

**Affiliations:** Duke University, Durham, North Carolina; Duke University, Durham, North Carolina; Dana-Farber Cancer Institute, Boston, Massachusetts; Duke University, Durham, North Carolina; Duke University Medical Center, Durham, North Carolina; University of Miami, Miami, Florida; Duke University Medical Center, Durham, North Carolina; University of Miami, Miami, Florida; Duke University, Durham, North Carolina; Duke University Medical Center, Durham, North Carolina; Duke University, Durham, North Carolina

## Abstract

**Background:**

The impact of host genetic variability on the outcome of S. aureus infection is unknown. HLA variation has evolved to ensure broad presentation of pathogen derived peptides to initiate protective adaptive immune responses. In genome-wide association studies we identified specific HLA class II variants (HLA-DRB1*04:01 & 04:02) that are positively associated with S. aureus infection. The goal of this study was to determine how HLA-DR variants may differ in their ability to bind and present S. aureus peptides to host T cells and impact protective immunity.

**Methods:**

*S. aureus* (USA300) proteasome was obtained (UP000008816) and a dataset of 468,812 overlapping 15 amino acid peptides was created. Peptide binding predictions for seven HLA variant subgroups were performed using a machine learning framework (MixMHC2pred 2.0). Binding prediction score of ≤ 2.0 was used as a biological threshold and data was analyzed using R (4.2.2). HLA-DR expression was measured on HLA homozygous B cell lines using flow cytometry (BD Fortessa) and antibodies specific for HLA-DR (L243 APC) and CD69 (FN50-PE) and analyzed using FlowJo and Microsoft Excel.

**Figure:**
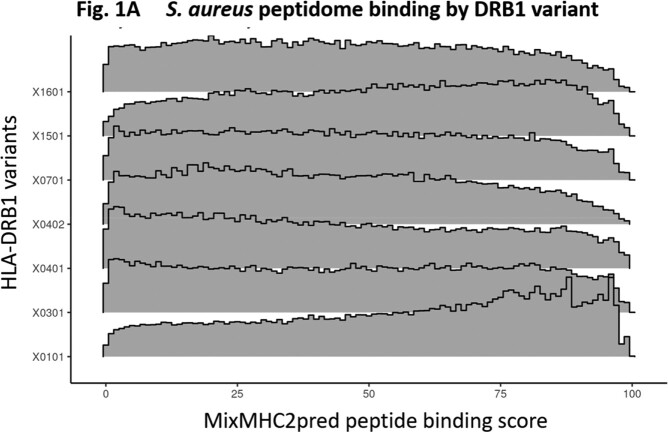

**Results:**

We observed different *S. aureus* peptide binding score distributions across HLA-DRB1 alleles representing unique peptide binding pockets (Fig 1A). Analysis revealed significantly higher proportions of peptides predicted to bind HLA-DRB1*04:01 and DRB1*04:02 compared to controls DRB1*15:01 and DRB1*01:01 (score ≤ 2.0). (Fig 1B). HLA-DR alleles also showed variable expression on HLA homozygous B cell lines, with DRB1*04:02 showing highest and DRB1*04:01 showing lowest expression (Fig 1C). HLA-DR expression was higher on B cells expressing activation marker CD69+ (p< 0.002) for all alleles except DRB1*04:01. *In vitro* T cell stimulation assays controlling for HLA genotype are in progress.

**Figure:**
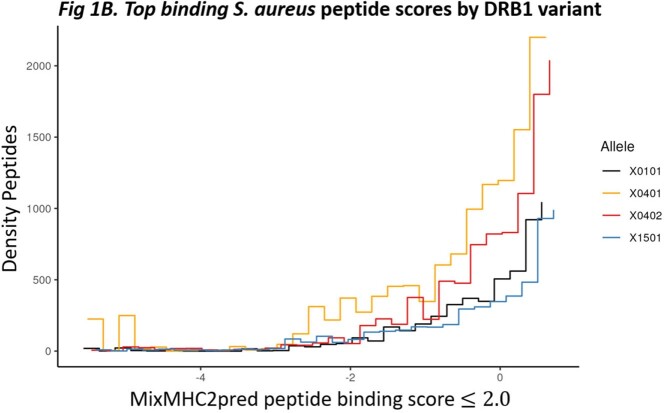

**Figure:**
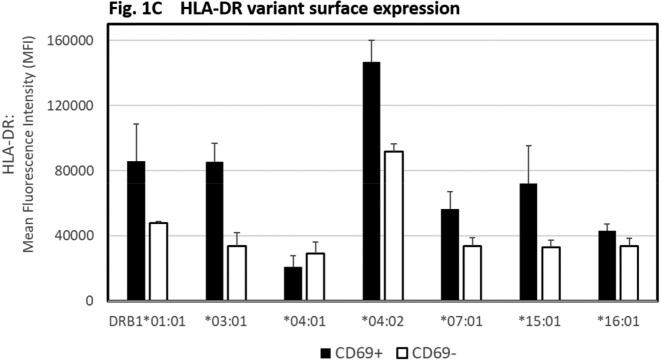

**Conclusion:**

We provide evidence to suggest that variation in peptide binding and cell surface expression may explain the association of HLA class II haplotypes with S. aureus infection. Elucidating the genetic basis of susceptibility to *S. aureus* infection will improve our understanding, treatment, and prevention of this urgent unmet medical need.

**Disclosures:**

**Vance G. Fowler, MD, MHS**, Amphliphi Biosciences, Integrated Biotherapeutics; C3J, Armata, Valanbio; Akagera, Aridis, Roche, Astra Zeneca: Advisor/Consultant|Genentech, Regeneron, Deep Blue, Basilea, Janssen;: Grant/Research Support|Infectious Diseases Society of America: Honoraria|MedImmune, Allergan, Pfizer, Advanced Liquid Logics, Theravance, Novartis, Merck; Medical Biosurfaces; Locus; Affinergy; Contrafect; Karius;: Grant/Research Support|Novartis, Debiopharm, Genentech, Achaogen, Affinium, Medicines Co., MedImmune, Bayer, Basilea, Affinergy, Janssen, Contrafect, Regeneron, Destiny,: Advisor/Consultant|Sepsis diagnostic: Patent pending|UpToDate: Royalties|Valanbio and ArcBio: Stock Options

